# Association between the oxidative balance score and diabetic kidney disease in diabetes mellitus patients: insights from NHANES 2011–2018

**DOI:** 10.3389/fnut.2025.1703451

**Published:** 2026-01-05

**Authors:** Qiuhong Li, Liuwei Wang, Zijun Yang, Yulin Wang, Lu Yu, Yanhong Guo, Zihan Zhai, Yan Liang, Dongxu Song, Lin Tang

**Affiliations:** Department of Nephrology, The First Affiliated Hospital of Zhengzhou University, Zhengzhou, Henan, China

**Keywords:** oxidative balance score, diabetic kidney disease, diabetes mellitus, dietary, lifestyle

## Abstract

**Background:**

Oxidative stress plays a crucial role in the onset and progression of diabetic kidney disease (DKD). The oxidative balance score (OBS) evaluates an individual’s dietary and lifestyle exposures related to oxidative stress. However, the association between the OBS and DKD remains unclear. This study aimed to investigate this association in patients with diabetes mellitus (DM).

**Methods:**

This cross-sectional study included 1,882 participants, representing 19.5 million individuals with DM, from the National Health and Nutrition Examination Survey (NHANES) collected between 2011 and 2018. The OBS was calculated using 20 dietary and lifestyle factors. DKD was defined as impaired glomerular filtration rate (estimated glomerular filtration rate [eGFR] < 60 mL/min/1.73 m^2^), albuminuria (urinary albumin-to-creatinine ratio [ACR] ≥ 30 mg/g), or both in DM patients. The association between the OBS and DKD was examined using weighted logistic regression and subgroup analyses.

**Results:**

The OBS was negatively associated with DKD. The multivariable-adjusted odds ratio (OR) for DKD per unit increase in the OBS as a continuous variable was 0.92 (95%CI: 0.85–0.99). When analyzed as a categorical variable, participants in the highest OBS quartile had significantly lower odds of DKD (OR: 0.26, 95%CI: 0.07–0.98) than those in the lowest quartile (*p* < 0.05). Further analyses revealed that the dietary OBS, but not lifestyle OBS, was significantly associated with DKD. The OBS had significant correlations with DKD among male patients. Among dietary components, fiber, carotene, niacin, vitamin C, calcium, and magnesium were the most strongly associated with lower odds of DKD.

**Conclusion:**

Our findings revealed a significant negative association between OBS levels and the presence of DKD in DM patients, suggesting that a higher antioxidant-rich diet score is associated with a lower likelihood of DKD. Future prospective studies are needed to confirm whether adopting such a lifestyle could serve as a strategy for DKD prevention.

## Background

Diabetic kidney disease (DKD), a primary complication of type 1 (T1DM) and type 2 (T2DM) diabetes (1), is the leading cause of end-stage renal disease (ESRD). The International Diabetes Federation projects a 46% increase in diabetes cases by 2045 compared to 2021 (2), with approximately one-third of these individuals expected to develop DKD. The prevention and delay of the progression of DKD have become the focus of current research.

The pathogenesis of DKD involves multiple factors, including glomerular hemodynamic alterations, overactivation of the renin–angiotensin–aldosterone system, inflammation, oxidative stress, endothelial cell damage, and mitochondrial dysfunction (3). Although the pathogenesis of DKD is multifactorial, the precise pathophysiological mechanisms remain unclear. Oxidative stress plays a crucial role in the development of DKD (4, 5) by disrupting the balance between oxidants and antioxidants (6). In a high glucose environment, oxidative stress can mediate mesangial cell proliferation, increase extracellular matrix secretion, and increase vascular permeability through various pathways, ultimately leading to the characteristic features of DKD, including extracellular matrix accumulation, glomerular basement membrane thickening, and endothelial cell dysfunction (7–9). Studies have also shown that high glucose-induced oxidative stress promotes podocyte shedding and apoptosis, thereby damaging the glomerular basement membrane and resulting in proteinuria (10–12). Overall, these findings underscore the significant contribution of oxidative stress to DKD progression. Restoring the balance between oxidative stress and antioxidants may serve as a future drug target to better prevent and treat DKD.

The OBS is an integrative measure of an individual’s oxidative and antioxidant status determined by evaluating the dietary and lifestyle components that contribute to antioxidant and pro-oxidant levels. A higher OBS indicates that antioxidants are predominating over pro-oxidants. Previous studies have demonstrated the negative associations between the OBS and the prevalence of hypertension (13), diabetes (14), abdominal adiposity (15), and mortality (16, 17). Moreover, studies have revealed that a higher OBS is associated with a lower prevalence of chronic kidney disease (CKD) (18, 19) and a lower risk of progression to ESRD (20), even though OBS is inversely associated with a number of diseases. However, the relationship between the OBS and DKD is not yet fully understood.

We hypothesized that a higher OBS is associated with a lower prevalence of DKD in patients with DM. This study aimed to assess the association between the OBS and DKD in DM patients using NHANES data from 2011 to 2018 in the United States. This study might have significant implications for the prevention of DKD if the findings are confirmed.

## Subjects and methods

Between 2011 and 2018, data were obtained from the NHANES, a biennial cross-sectional study conducted by the National Center for Health Statistics (NCHS) to assess health and nutrition in adults and children.

Each year, approximately 5,000 participants are chosen randomly through a multistage, stratified probability sampling method. The participants were evaluated nutritionally and physically at mobile examination centers using standardized interviews, physical examinations, and laboratory tests. The NHANES program was approved by the NCHS Ethics Review Board (ERB), with participants in this survey providing written informed consent, and any details that might disclose their identity were withheld. There are free detailed statistics available at: https://www.cdc.gov/nchs/nhanes/.

**Figure 1:**
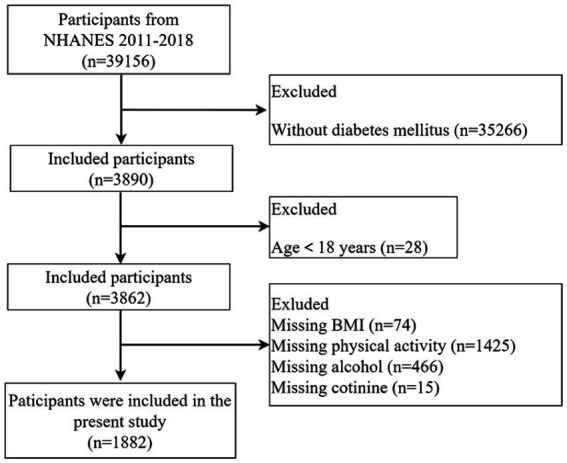
Flowchart of subject selection for this study.

From the 39,156 NHANES 2011–2018 participants, we excluded 35,266 participants without diabetes mellitus, 28 participants under 18 years, 3,840 lacking lifestyle OBS data, 74 with missing body mass index (BMI) data, 1,425 with missing physical activity data, 466 with missing alcohol data, and 15 with missing cotinine. Ultimately, the study enrolled 1,882 participants. The selection process is shown in Figure 1.

### Covariates

This study incorporated covariates potentially influencing the OBS–DKD relationship. The study included demographic variables such as sex, age, and ethnicity; physical examination measures such as systolic blood pressure, diastolic blood pressure, and BMI; laboratory tests including white blood cell, hemoglobin, blood platelet, urinary albumin creatinine ratio, blood urea nitrogen, serum creatinine, estimated glomerular filtration rate, serum uric acid, fasting blood glucose, plasma albumin, aspartate aminotransferase, alanine aminotransferase, glycated hemoglobin, plasma triglyceride, total cholesterol, low-density lipoprotein cholesterol (LDL-C), and high-density lipoprotein cholesterol (HDL-C); health risk factors including smoking status, drinking status, congestive heart failure (CHF), cardiovascular disease (CVD), and hypertension; and drug treatment including hypoglycemic drug use.

### OBS

The OBS was determined by aggregating the points allocated to each component, as outlined in prior research (21, 22). The OBS was divided into two categories: 16 dietary OBSs and 4 lifestyle OBSs, including 5 pro-oxidants and 15 antioxidants. In total, 16 of the dietary components included dietary fiber, carotene, riboflavin, niacin, vitamin B6, total folate, vitamin B12, vitamin C, vitamin E, calcium, magnesium, zinc, copper, total fat, and iron, and four of the lifestyle components included physical activity, BMI, alcohol, and cotinine. A higher OBS signifies increased antioxidant exposure. Cotinine, with its longer blood half-life, was used to assess smoking status, as it can measure both direct tobacco use and exposure to environmental tobacco smoke. Data on physical activity were sourced from the NHANES physical activity questionnaire (PAQ). Physical activity was measured as metabolic equivalent (MET) score, weekly frequency of each physical activity, and each physical activity’s duration according to previous studies (22, 23).

The OBS components were assigned according to the following scheme: non-drinkers received 2 points, non-heavy drinkers (0–15 g/day for female participants and 0–30 g/day for male participants) received 1 point, and heavy drinkers (≥15 g/day for female participants and ≥30 g/day for male participants) received 0 points. Subsequently, the remaining components were categorized into three groups based on sex-specific tertiles. In the case of antioxidants, scores ranged from 0 to 2 for groups across tertiles 1 to 3. For pro-oxidants, scores ranged from 2 to 0, respectively (Table 1).

**Table 1:** Ingredients that make up the oxidative balance score.

OBS components	Property	Male			Female		
		0	1	2	0	1	2
Dietary OBS components							
Dietary fiber (g/d)	A	<13.60	13.60–21.40	≥21.40	<11.30	11.30–17.45	≥17.45
Carotene (RE/d)	A	<406.00	406.00–724.00	≥724.00	<342.50	342.50–586.50	≥586.50
Riboflavin (mg/d)	A	<1.63	1.63–2.40	≥2.40	<1.23	1.23–1.78	≥1.78
Niacin (mg/d)	A	<20.70	20.70–29.67	≥29.67	<15.46	15.46–21.94	≥21.94
Vitamin B6 (mg/d)	A	<1.63	1.63–2.43	≥2.43	<1.23	1.23–1.79	≥1.79
Total folate (mcg/d)	A	<303.50	303.50–456.00	≥456.00	<240.50	240.50–353.50	≥353.50
Vitamin B12 (mcg/d)	A	<3.14	3.14–5.53	≥5.53	<2.27	2.27–3.97	≥3.97
Vitamin C (mg/d)	A	<43.30	43.30–89.25	≥89.25	<38.40	38.40–88.45	≥88.45
Vitamin E (ATE) (mg/d)	A	<6.22	6.22–9.87	≥9.87	<4.90	4.90–7.70	≥7.70
Calcium (mg/d)	A	<692.00	692.00–1,067.00	≥1,067.00	<557.00	557.00–888.00	≥888.00
Magnesium (mg/d)	A	<251.00	251.00–352.00	≥352.00	<199.50	199.50–277.50	≥277.50
Zinc (mg/d)	A	<9.04	9.04–13.26	≥13.26	<6.38	6.38–9.46	≥9.46
Copper (mg/d)	A	<0.99	0.99–1.39	≥1.39	<0.80	0.80–1.14	≥1.14
Selenium (mcg/d)	A	<96.80	96.80–138.15	≥138.15	<72.00	72.00–103.65	≥103.65
Total fat (g/d)	P	≥99.96	64.93–99.96	<64.93	≥74.78	46.81–74.78	<46.81
Iron (mg/d)	P	≥17.06	11.81–17.06	<11.81	≥13.59	9.19–13.59	<9.19
Lifestyle OBS components							
Physical activity (MET-hour/week)	A	<16.00	16.00–58.00	≥58.00	<12.00	12.00–34.67	≥12.00
Alcohol (g/d)	P	≥30	0–30	None	≥15	0–15	None
Body mass index (kg/m^2^)	P	≥32.60	27.80–32.60	<27.80	≥36.30	30.20–36.30	<30.20
Cotinine (ng/mL)	P	>0.12	0.02–0.12	≤0.02	>0.05	0.01–0.05	≤0.01

OBS, oxidative balance score; A, antioxidant; P, pro-oxidant; RE, retinol equivalent; ATE, alpha-tocopherol equivalent; MET, metabolic equivalent.

### Definition of covariates

The study defined diabetes as follows: (1) a self-reported physician diagnosis, (2) fasting plasma glucose greater than or equal to 7.0 mmol/L, (3) glycated hemoglobin (HbA1c) ≥ 6.5 mmol/L, or (4) taking diabetes medications. DKD was defined as eGFR [calculated using the chronic kidney disease epidemiology (CKD-EPI) formula] < 60 mL/min/1.73 m^2^ and/or ACR (≥30 mg/g) in patients with DM.

### Statistical analysis

As a result of the complex survey design, weighted analyses were conducted in accordance with NHANES recommendations. Baseline data for continuous variables were presented as mean ± standard deviation (SD) for approximately normal distributions and as median with interquartile range (IQR) for skewed distributions. Categorical variables were expressed as a number (percentage). To compare differences in baseline characteristics across groups divided by OBS quartiles, the weighted variance (analysis of variance [ANOVA]) or the Kruskal–Wallis test was used for continuous variables, and the weighted chi-squared (*χ*^2^) test was used for categorical variables. A sampling-weighted multivariate logistic regression model was used to analyze the relationship between the OBS and DKD, with results expressed as odds ratios (ORs) and 95% confidence intervals (95% CI). Subgroup analyses were performed to identify factors potentially affecting the stability of the findings. We used the least absolute shrinkage and selection operator (LASSO) regression model to address collinearity among variables and pinpoint key dietary and lifestyle factors influencing DKD, choosing the model with the minimum deviance lambda value.

Statistical analyses were conducted using R software (version 4.3.2) and GraphPad Prism (version 8.0, San Diego, CA, USA). Two-tailed *p*-values of <0.05 were considered statistically significant.

## Results

### Baseline characteristics

A total of 1,882 participants with DM, representing 19,485,128.9 adults with DM in the US, were included in our study. Among them, DKD patients accounted for approximately 31.9%. Characteristics of the sample with weighted participant numbers stratified by OBS quartiles are shown in Table 2. At baseline, 55.15% of the participants (representing 10.7 million US adults) were male, and 44.85% of the participants (representing 8.7 million US adults) were female. Participants with high OBS scores tended to have a higher level of eGFR, ALB, and HDL-C and a lower level of BMI, white blood cell (WBC), ACR, serum creatinine (SCR), blood urea nitrogen (BUN), serum uric acid (SUA), and total cholesterol (TC). They were also less likely to have CHF, CVD, hypertension, or DKD.

**Table 2:** Baseline characteristics of participants according to the oxidative balance score’s quartiles.

Characteristic	OBS quartiles				*p*-value
	Quartile 1	Quartile 2	Quartile 3	Quartile 4	
*N*	4,538,980.7	4,878,257.3	5,346,899.6	4,720,991.3	
Demographics					
Male, *n* (%)	2,264,481.1 (49.9)	2,856,634.8 (58.6)	3,096,302.0 (57.9)	2,529,598.0 (53.6)	0.328
Age (y)	59.00 (49.00, 69.00)	59.00 (50.00, 67.00)	60.45 (50.00, 70.00)	57.29 (49.66, 67.00)	0.684
Ethnicity, *n* (%)					
Mexican American	363,994.2 (8.0)	549,685.0 (11.3)	582,977.9 (10.9)	564,632.3 (12.0)	0.012
Other Hispanic	331,298.2 (7.3)	275,849.7 (5.7)	354,064.6 (6.6)	324,013.4 (6.9)	
Non-Hispanic White	2,562,557.6 (56.5)	2,944,525.6 (60.4)	3,106,259.1 (58.1)	2,844,543.7 (60.3)	
Non-Hispanic Black	946,845.6 (20.9)	715,101.2 (14.7)	670,776.9 (12.5)	420,883.7 (8.9)	
Other ethnicities	334,285.1 (7.4)	393,095.9 (8.1)	632,821.1 (11.8)	566,918.1 (12.0)	
Physical characteristics					
SBP (mmHg)	128.67 (116.67, 139.64)	126.67 (117.33, 140.00)	128.67 (119.33, 138.67)	125.33 (115.33, 137.19)	0.204
DBP (mmHg)	70.00 (62.22, 76.16)	72.00 (63.33, 78.67)	70.67 (63.33, 77.68)	71.33 (64.67, 79.33)	0.311
BMI (kg/m^2^)	32.90 (28.83, 37.60)	31.70 (28.70, 35.50)	31.70 (27.90, 36.70)	30.32 (27.30, 35.40)	0.112
Biochemical measurements					
WBC (*10^9^/L)	7.50 (6.40, 9.00)	7.60 (6.20, 8.98)	7.60 (6.40, 9.00)	7.20 (5.90, 8.70)	0.115
HGB (g/dL)	13.90 (12.80, 15.00)	14.50 (13.40, 15.20)	14.30 (13.20, 15.20)	14.20 (13.50, 15.10)	0.009
PLT (*10^9^/L)	228.00 (183.00, 287.00)	225.00 (192.00, 268.00)	219.85 (182.00, 271.00)	224.00 (191.00, 256.94)	0.832
ACR (mg/g)	12.68 (7.06, 33.86)	9.60 (5.99, 28.14)	9.91 (6.11, 21.04)	10.21 (5.42, 22.48)	0.184
BUN (mmol/L)	4.64 (3.93, 6.43)	5.36 (4.27, 6.78)	5.00 (4.28, 6.48)	5.36 (4.28, 6.78)	0.029
SCR (umol/L)	78.42 (62.76, 104.31)	78.68 (64.53, 91.94)	77.73 (62.76, 92.82)	75.14 (63.65, 89.47)	0.226
eGFR (mL/min/1.73 m^2^)	86.64 (58.17, 104.97)	90.36 (70.79, 102.33)	88.27 (69.39, 103.19)	88.69 (73.81, 100.85)	0.541
SUA (umol/L)	350.90 (291.50, 416.40)	327.10 (279.60, 380.70)	327.10 (273.60, 398.50)	315.20 (267.70, 374.70)	0.012
FBG (mmol/L)	7.43 (6.57, 9.45)	7.52 (6.77, 9.47)	7.72 (7.05, 9.49)	7.59 (6.79, 9.60)	0.871
ALT (U/L)	21.00 (15.00, 28.00)	24.00 (18.00, 34.00)	24.48 (18.00, 31.00)	23.00 (18.00, 31.00)	0.021
AST (U/L)	22.00 (18.00, 29.00)	22.00 (18.00, 28.00)	23.00 (19.09, 29.00)	22.00 (18.00, 29.00)	0.518
ALB (g/L)	41.00 (38.00, 43.00)	42.00 (40.00, 45.00)	42.00 (40.00, 44.00)	42.00 (40.00, 44.00)	0.009
TC (mmol/L)	4.60 (3.93, 5.38)	4.64 (3.88, 5.48)	4.55 (3.84, 5.25)	4.63 (3.83, 5.37)	0.577
HDL-C (mmol/L)	1.14 (0.98, 1.32)	1.16 (0.98, 1.40)	1.19 (0.98, 1.38)	1.14 (0.98, 1.37)	0.541
LDL-C (mmol/L)	2.42 (2.02, 3.13)	2.85 (1.99, 3.39)	2.59 (1.94, 3.13)	2.59 (1.94, 3.21)	0.513
TG (mmol/L)	1.63 (1.13, 2.09)	1.35 (1.00, 1.93)	1.43 (0.88, 2.10)	1.55 (1.00, 2.40)	0.111
HbA1c (%)	6.70 (6.10, 7.90)	6.70 (6.10, 7.60)	6.60 (6.20, 7.60)	6.70 (6.00, 7.70)	0.970
Medication					
Hypoglycemic drug use, *n* (%)	2,440,626.6 (61.2)	2,745,479.2 (65.0)	3,300,511.0 (71.3)	2,568,526.6 (61.7)	0.213
Risk factors					
Smoking status, *n* (%)	765,134.2 (31.0)	765,527.2 (34.3)	599,309.9 (23.7)	435,272.4 (21.3)	0.225
Drinking status, *n* (%)	3,323,160.6 (75.0)	3,765,748.5 (78.8)	4,080,510.9 (77.9)	3,709,148.6 (79.7)	0.663
Health conditions					
CHF, *n* (%)	370,677.3 (8.2)	346,890.8 (7.1)	294,911.3 (5.5)	238,296.3 (5.1)	0.575
CVD, *n* (%)	523,582.0 (11.6)	539,685.0 (11.2)	592,553.9 (11.2)	393,293.5 (8.3)	0.795
DKD, *n* (%)	1,871,613.6 (41.2)	1,549,351.0 (31.8)	1,529,222.0 (28.6)	1,266,119.4 (26.8)	0.013

OBS, oxidative balance score; SBP, systolic blood pressure; DBP, diastolic blood pressure; BMI, body mass index; WBC, white blood cell; HGB, hemoglobin; PLT, blood platelet; ACR, urinary albumin-to-creatinine ratio; FBG, fasting blood glucose; HbA1c, glycated hemoglobin; TG, plasma triglyceride; TC, total cholesterol; LDL-C, low-density lipoprotein cholesterol; HDL-C, high-density lipoprotein cholesterol; BUN, blood urea nitrogen; SCR, serum creatinine; eGFR, estimated glomerular filtration rate; SUA, serum uric acid; ALB, plasma albumin; AST, aspartate aminotransferase; ALT, alanine aminotransferase; CHF, congestive heart failure; CVD, cardiovascular disease; DKD, diabetic kidney disease.

Data are presented as the mean ± standard error or median (interquartile range) for continuous variables and the numbers (percentage) for categorical variables.

*p* < 0.05 was considered statistically significant.

### Relationship between the OBS and DKD

A sampling-weighted multivariate logistic regression analysis revealed an association between the OBS and DKD and found that a lower OBS was correlated with DKD in patients with DM (Table 3). After adjusting for age, sex, ethnicity, HGB, ACR, SBP, DBP, HbA1c, ALT, AST, eGFR, TC, smoking status, drinking status, CHF, CVD, and hypoglycemic drug use (model 3), the association was also significant. The adjusted OR for DKD per unit increase in the OBS as a continuous variable was 0.92 (95% CI: 0.85–0.99). When the OBS was analyzed as a categorical variable, the ORs (95% CIs) for DKD in the Quartile 1, Quartile 2, Quartile 3 of the OBS were 0.16 (0.06–0.46), 0.13 (0.03–0.56), and 0.26 (0.07–0.98) compared with quartile 1, respectively (all *p* < 0.05).

**Table 3:** Relationship between the oxidative balance score and DKD.

Variables	Model 1		Model 2		Model 3	
	OR (95% CI)	*p*-value	OR (95% CI)	*p*-value	OR (95% CI)	*p*-value
OBS	0.97 (0.95–0.99)	<0.001	0.97 (0.95–0.98)	<0.001	0.92 (0.85–0.99)	0.028
Quartiles of OBS		0.005		0.002		0.002
Quartile 1	Reference		Reference		Reference	
Quartile 2	0.66 (0.42–1.05)		0.66 (0.40–1.07)		0.16 (0.06–0.46)	
Quartile 3	0.57 (0.37–0.89)		0.53 (0.34–0.82)		0.13 (0.03–0.56)	
Quartile 4	0.52 (0.36–0.76)		0.51 (0.35–0.75)		0.26 (0.07–0.98)	

CI, confidence interval; OR, odds ratio.

Model 1: no adjustment; Model 2: adjusted for age and sex.

Model 3: adjusted for all the factors in model 1 and ethnicity, HGB, ACR, SBP, DBP, HbA1c, ALT, AST, eGFR, TC, smoking status, drinking status, CHF, CVD, and hypoglycemic drug use at baseline.

### Relationship between the dietary/lifestyle OBSs and DKD

Table 4 presents the findings from sampling-weighted multivariate logistic regression analyses, highlighting the associations between the dietary and lifestyle OBSs and DKD. The association between the dietary OBS and DKD was statistically significant (all *p* < 0.05), with ORs (95% CIs) of 0.90 (0.83–0.98) when dietary OBS was analyzed as a continuous variable and with ORs (95% CIs) for the upper quartile of the dietary OBSs of 0.10 (0.03–0.29), 0.16 (0.04–0.61), and 0.18 (0.04–0.73) compared to the lowest quartile, respectively, when the dietary OBS is a categorical variable after adjusting for age, sex, ethnicity, HGB, ACR, SBP, DBP, HbA1c, ALT, AST, eGFR, TC, smoking status, drinking status, CHF, CVD, and hypoglycemic drug use. The association between the lifestyle OBS and DKD was not statistically significant (all *p* > 0.05). The OR (95% CI) for the lifestyle OBS as a continuous variable was 1.31 (0.90–1.91). When categorized, the ORs (95% CIs) for the upper quartiles were 0.72 (0.19–2.76), 2.01 (0.62–6.57), and 0.67 (0.12–3.62), respectively, compared to the lowest quartile.

**Table 4:** Association between the dietary/lifestyle OBS with DKD.

Variables	Model 1		Model 2		Model 3	
	OR (95% CI)	*p*-value	OR (95% CI)	*p*-value	OR (95% CI)	*p*-value
Dietary OBS	0.96 (0.95–0.98)	<0.001	0.96 (0.94–0.98)	<0.001	0.90 (0.83–0.98)	0.014
Quartiles of the dietary OBS		0.001		0.002		<0.001
Quartile 1	Reference		Reference		Reference	
Quartile 2	0.53 (0.35–0.81)		0.51 (0.33–0.80)		0.10 (0.03–0.29)	
Quartile 3	0.56 (0.36–0.86)		0.53 (0.34–0.82)		0.16 (0.04–0.61)	
Quartile 4	0.48 (0.32–0.72)		0.49 (0.32–0.76)		0.18 (0.04–0.73)	
Lifestyle OBS	1.00 (0.89–1.12)	>0.900	0.95 (0.85–1.06)	0.400	1.31 (0.90–1.91)	0.140
Quartiles of lifestyle OBS		0.200		0.300		0.400
Quartile 1	Reference		Reference		Reference	
Quartile 2	1.11 (0.70–1.77)		0.93 (0.59–1.49)		0.72 (0.19–2.76)	
Quartile 3	1.51 (0.91–2.52)		1.24 (0.73–2.10)		2.01 (0.62–6.57)	
Quartile 4	0.82 (0.50–1.35)		0.67 (0.40–1.11)		0.67 (0.12–3.62)	

CI, confidence interval; OR: odds ratio.

Model 1: no adjustment; Model 2: adjusted for age and sex.

Model 3: adjusted for all the factors in model 1 and ethnicity, HGB, ACR, SBP, DBP, HbA1c, ALT, AST, eGFR, TC, smoking status, drinking status, CHF, CVD, and hypoglycemic drug use at baseline.

### Subgroup analyses

**Figure 2:**
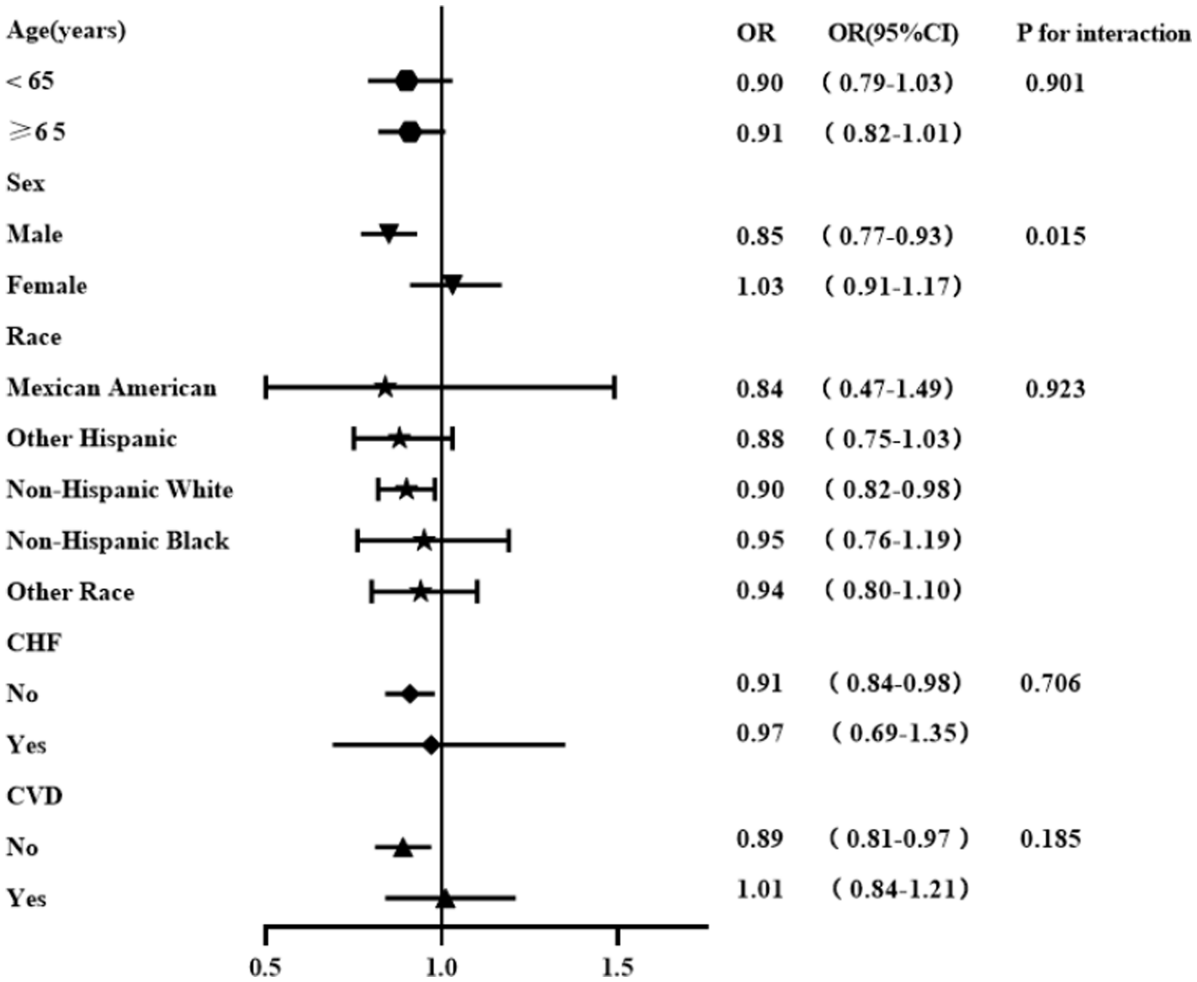
Stratified analyses of the relationship between the OBS and DKD. Adjusted for age, sex, ethnicity, HGB, ACR, SBP, DBP, HbA1c, ALT, AST, eGFR, TC, smoking status, drinking status, CHF, CVD, and hypoglycemic drug use at baseline.

We analyzed the associations between the OBS and DKD, stratified by age, sex, ethnicity, CHF, and CVD (Figure 2). No significant interactions were observed for age, ethnicity, smoking status, CHF, or CVD (P for interaction > 0.05 for all). However, the OBS showed a significant correlation with DKD in male patients (*p* for interaction = 0.015). The inverse association between the OBS and DKD was more pronounced and statistically significant in male patients (OR: 0.85, 95% CI: 0.77–0.93) than in female patients (OR: 1.03, 95% CI: 0.91–1.17).

### Identification of key DKD-related OBS factors

**Figure 3:**
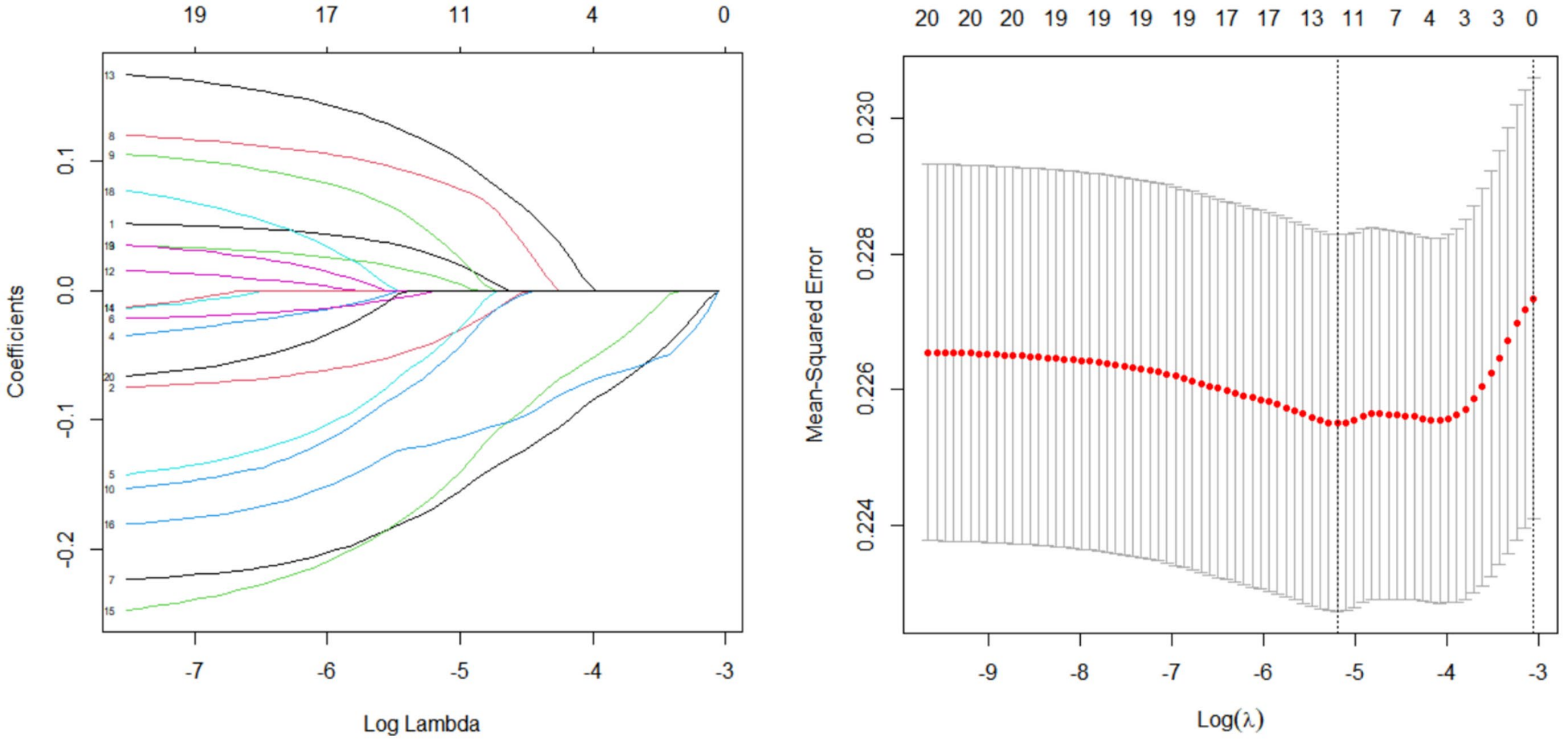
LASSO penalized regression analysis for identifying key oxidative balance factors associated with DKD. All components were standardized (mean = 0, SD = 1) prior to the LASSO regression analysis. The optimal penalty parameter (*λ*) was selected via 10-fold cross-validation using the minimum criterion.

To identify the specific dietary components most strongly associated with DKD, a LASSO penalized regression model was applied to the 16 dietary OBS components. The LASSO regression model aims to identify key parameters by incorporating an L1 regularization term into ordinary least squares regression. This approach helps prevent overfitting and ensures the selection of appropriate factors, particularly when multiple factors are correlated. Dietary fiber, carotene, niacin, vitamin C, calcium, and magnesium were identified as the six dietary factors most closely associated with DKD (Figure 3).

## Discussion

In this nationally representative study of U.S. adults with DM, we found that a higher OBS, indicative of a predominance of antioxidant exposures, was significantly associated with a lower prevalence of DKD. Further analyses indicated that the dietary OBS was notably associated with a lower prevalence of DKD. Additionally, the OBS showed significant correlations with DKD among male patients. Moreover, our study identified 6 factors from a total of 16 dietary OBS components that were most closely associated with DKD. These factors include dietary fiber, carotene, niacin, vitamin C, calcium, and magnesium. Understanding the factors associated with DKD prevalence may help identify potential targets for future interventions aimed at mitigating complications and enhancing patient quality of life.

Our findings align with and substantially extend previous studies that a higher OBS may be independently associated with a lower prevalence of CKD in the general population and DKD in individuals with T2DM (18, 19, 24, 25). However, our study specifically focuses on the high-risk DM population and conducts further statistical analyses, providing more targeted evidence. Research indicates that the OBS effectively evaluates oxidative balance (26–30), while the weighted OBS is more suitable for assessing the balance between antioxidants and pro-oxidants (31). The association between oxidative stress and DKD is complex and significant, driven by hyperglycemia-induced overproduction of mitochondrial ROS, which activates multiple pathological pathways, including Protein Kinases C (PKC), Advanced Glycation End-products (AGEs), and hexosamine biosynthetic flux, ultimately leading to renal inflammation, fibrosis, and apoptosis (32). Our results provide epidemiological support for the concept that counteracting this oxidative stress with antioxidant-rich diets can confer renal protection. This perspective is strongly supported by recent evidence demonstrating that natural antioxidants and phytochemicals—such as carotenoids, niacin (and its metabolites), and vitamin C—mitigate diabetic complications by modulating key signaling pathways including Nrf2 and SIRT1, which regulate cellular antioxidant defenses and mitochondrial function (33, 34). For instance, a recent review comprehensively outlines the beneficial effects of natural antioxidants on oxidative stress-mediated diabetes complications (35), directly reinforcing the biological plausibility of our finding. Notably, the ORs for OBSs in Model 3 of our logistic regression analyses are lower than those previously reported (24, 25). We believe that this discrepancy is largely attributable to our additional adjustment for baseline eGFR and ACR in the multivariable models, as these two parameters are central to the diagnosis of DKD. This finding not only confirms the robust relationship between the OBS and DKD but also suggests that the protective mechanisms may partly operate through the preservation of renal function, while also involving direct pathways independent of current renal function.

Consistent with previous studies, the subgroup analysis revealed that sex significantly influenced the associations between the OBS and DKD. This variation may be explained by the differing roles of sex hormones in oxidative stress. Estrogens, for instance, possess antioxidant properties and can upregulate Nrf2 signaling, potentially providing premenopausal women with baseline protection that attenuates the additional benefit of dietary antioxidants measured by the OBS. In contrast, the relative deficiency of these protective effects in male individuals might render them more susceptible to the modulatory impact of dietary antioxidant intake, leading to a more pronounced observed association. Future research should delve deeper into the mechanisms behind the sex difference in antioxidant lifestyle interventions.

A pivotal advancement of our study is the application of LASSO regression, which moves beyond the composite score to pinpoint specific dietary factors. We found that dietary fiber, carotene, niacin, vitamin C, calcium, and magnesium are more closely associated with the prevalence of DKD in DM. The synergistic action of these nutrients likely contributes to the observed renal protection. Dietary fiber, through its fermentation into short-chain fatty acids, shapes gut microbiota and reduces systemic inflammation (36–38). Carotene and vitamin C are potent direct free radical scavengers that also support the regeneration of other antioxidants (39, 40). Niacin and its metabolite nicotinamide are precursors for NAD+, a cofactor essential for SIRT1 deacetylase activity, which improves mitochondrial efficiency and suppresses oxidative stress (41). Furthermore, calcium and magnesium are crucial for maintaining ionic homeostasis and proper mitochondrial function, whose dysregulation is implicated in oxidative stress in DKD (42, 43). Therefore, due to various biological contributions to different diseases, the identified combination of nutrients appears to be particularly relevant for DKD pathophysiology. Future research should prioritize exploring the synergistic effects of these specific nutrients.

There is a significant association between dietary OBS and DKD, rather than the lifestyle OBS, indicating that nutrient intake may play a more significant role than lifestyle factors in the context of DKD association (44). The reason may be that the relationship between lifestyle factors (such as BMI and cotinine) and renal function is often non-linear and complex (45, 46), which might explain their attenuated role within the overall OBS framework in our cohort. However, overall, the OBS was a measure of the body’s overall oxidation/antioxidative balance, potentially providing a more accurate evaluation of oxidative stress than individual components, which may not completely reveal antioxidant mechanisms. From a clinical translational perspective, the OBS, derived from diet and lifestyle questionnaires, offers a safe and cost-effective tool for assessing oxidative stress balance. It could be integrated into clinical practice to guide nutritional interventions for preventing DKD. For instance, clinicians could use a simplified OBS assessment to identify patients with low antioxidant intake and provide targeted counseling to increase the consumption of foods rich in the six key nutrients we identified.

The study possesses multiple strengths. First, a nationally representative NHANES population was selected as the survey object, and the data were weighted to ensure the generalizability of the findings. Second, we adjusted for confounding factors in the analyses. Third, we analyzed the separate impacts of dietary and lifestyle OBSs on DKD and identified significant components within the dietary OBS using an advanced statistical approach (LASSO) to perform an exploratory analysis.

There are also several limitations. This cross-sectional study establishes the relationship between the OBS and DKD but does not allow for causal inference. Second, although we adjusted for several confounders, we could not fully account for the effects of all medications, such as statins, antihypertensives (particularly angiotensin-converting-enzyme [ACE] inhibitors/angiotensin receptor blockers [ARBs]), and antioxidant supplements that might influence the body’s oxidative balance.

## Conclusion

There is a significantly negative correlation between the OBS and DKD, especially dietary OBS. These findings highlight the association of a high-quality diet with a lower prevalence of DKD in patients with DM, suggesting a potential link through the reduction of inflammation and oxidative stress. These provide a foundation for future research aimed at developing targeted dietary recommendations for diabetes patients at risk of DKD. Future research should investigate the causal relationship and the precise mechanism connecting the OBS and DKD.

## Data Availability

The original contributions presented in the study are included in the article/supplementary material; further inquiries can be directed to the corresponding author.
